# Validation of risk scores for prediction of severe pneumonia in kidney transplant recipients hospitalized with community-acquired pneumonia

**DOI:** 10.1007/s15010-023-02101-z

**Published:** 2023-11-20

**Authors:** Moritz Müller-Plathe, Bilgin Osmanodja, Georg Barthel, Klemens Budde, Kai-Uwe Eckardt, Martin Kolditz, Martin Witzenrath

**Affiliations:** 1 Department of Infectious Diseases, Respiratory Medicine and Critical Care, Charité–Universitätsmedizin Berlin, Corporate Member of Freie Universität Berlin, Humboldt-Universität zu Berlin, Berlin, Germany; 2 Department of Nephrology and Medical Intensive Care, Charité–Universitätsmedizin Berlin, Corporate Member of Freie Universität Berlin, Humboldt-Universität zu Berlin, Berlin, Germany; 3 Department of Anesthesiology and Operative Intensive Care Medicine, Charité–Universitätsmedizin Berlin, Corporate Member of Freie Universität Berlin, Humboldt-Universität zu Berlin, Berlin, Germany; 4 Division of Pulmonology, Medical Department I, University Hospital Carl Gustav Carus, Technische Universität Dresden, Dresden, Germany

**Keywords:** CAP, Severe pneumonia, Risk prediction, Kidney transplantation, Immunosuppression

## Abstract

**Purpose:**

Risk scores for community-acquired pneumonia (CAP) are widely used for standardized assessment in immunocompetent patients and to identify patients at risk for severe pneumonia and death. In immunocompromised patients, the prognostic value of pneumonia-specific risk scores seems to be reduced, but evidence is limited. The value of different pneumonia risk scores in kidney transplant recipients (KTR) is not known.

**Methods:**

Therefore, we retrospectively analyzed 310 first CAP episodes after kidney transplantation in 310 KTR. We assessed clinical outcomes and validated eight different risk scores (CRB-65, CURB-65, DS-CRB-65, qSOFA, SOFA, PSI, IDSA/ATS minor criteria, NEWS-2) for the prognosis of severe pneumonia and in-hospital mortality. Risk scores were assessed up to 48 h after admission, but always before an endpoint occurred. Multiple imputation was performed to handle missing values.

**Results:**

In total, 16 out of 310 patients (5.2%) died, and 48 (15.5%) developed severe pneumonia. Based on ROC analysis, sequential organ failure assessment (SOFA) and national early warning score 2 (NEWS-2) performed best, predicting severe pneumonia with AUC of 0.823 (0.747–0.880) and 0.784 (0.691–0.855), respectively.

**Conclusion:**

SOFA and NEWS-2 are best suited to identify KTR at risk for the development of severe CAP. In contrast to immunocompetent patients, CRB-65 should not be used to guide outpatient treatment in KTR, since there is a 7% risk for the development of severe pneumonia even in patients with a score of zero.

**Supplementary Information:**

The online version contains supplementary material available at 10.1007/s15010-023-02101-z.

## Introduction

With therapy regimens consisting regularly of two or more immunosuppressive agents, kidney transplant recipients (KTR) are prone to severe infectious complications [1]. Septicemia and pneumonia are among the ten most frequent causes for admission to the emergency department in KTR [2], while pneumonia is the most common life-threatening infection in KTR [3, 4]. In fact, due to improved immunosuppressive regimens and increased life expectancy, the number of KTR presenting with community-acquired pneumonia (CAP) is increasing constantly [5].

Characterizing this population of patients and identifying KTR at risk for severe CAP or death through risk scores is crucial to improve initial management and patient outcomes. However, management recommendations for immunocompromised patients with CAP mostly rely on expert consensus statements since these patients have so far usually been excluded from national guidelines [6–8]. Even though new guidelines for solid organ recipients with CAP were published in 2019, risk stratification in these patients remains difficult due to lack of data from clinical trials [9].

Pneumonia-specific risk scores such as CRB-65 [10], CURB-65 [11], DS-CRB-65 [12], PSI (pneumonia severity index) [13], and IDSA/ATS (Infectious Diseases Society of America)/ATS (American Thoracic Society) minor criteria [14] are well established in immunocompetent patients presenting with CAP. Moreover, qSOFA (quick sequential organ failure assessment) [15] and SOFA (sequential organ failure assessment) [16], initially developed to predict sepsis outcome, have been used more frequently to predict CAP severity [17–19]. NEWS-2 (national early warning score 2) is the currently recommended score for determining the degree of illness of a patient by the National Health Service (NHS) [20].

In different cohorts of immunocompromised patients with CAP, the prognostic value of CRB-65 and qSOFA was found to be limited. Carrabba et al. showed poor prognostic value of C(U)RB-65 and PSI in patients with immunosuppression (AUC for mortality between 0.55 and 0.64) [21]. Frantz et al. found comparable results in a cohort of 198 immunocompromised patients (AUC for severe ***CAP-CRB-65: 0.63 and qSOFA: 0.69) [19]. While the first cohort did not include any solid organ transplant recipients, the latter included only 18 KTR. In summary, there are no enough data to recommend for or against using risk scores and to choose among them in KTR presenting with CAP.

Therefore, we characterized a cohort of 390 KTR with CAP at our tertiary care center and compared the validity of eight different risk scores for prediction of in-hospital mortality and severe CAP analyzing 310 first CAP episodes in 310 KTR.

## Methods

### Study population

The study was approved by the ethics committee of the Charité–Universitätsmedizin Berlin (EA1/330/21). We screened our proprietary electronic health record and transplant database TBase [22] for patients with pneumonia, who were treated at Charité–Universitätsmedizin Berlin between 01.01.2006 and 31.03.2022, were at least 18 years old and had a functioning kidney transplant at the time of diagnosis as detailed in Item S1. Next, we reviewed all medical records of the respective 1103 medical cases with suspected CAP to include only patients meeting the CAP definition and none of the exclusion criteria as shown in Table 1, as well as to extract demographic and clinical data detailed in Item S2. The main analysis was performed for the first pneumonia for each patient in our records to ensure statistical independence. Subsequently, we analyzed the recurrent cases in patients with more than one CAP episode.

**Table 1:** Inclusion and exclusion criteria

Inclusion criteria
Patient 18 years or older at the time of hospital admission
Functioning kidney transplant at the time of hospital admission
Community-acquired pneumonia
Pneumonia: pneumonia-like consolidation on CT/Chest X-Ray + at least one of the following 5 criteria in the first 2 days)
New onset of cough
Purulent sputum
Cracklings on auscultation
Fever—temperature > 37.8 °C (rectal) or a temperature of > 38.3 °C (axillar, oral, auricular, or sublingual)
Shortness of breath as defined by tachypnea, dyspnea, or hypoxemia (novel oxygen supplementation, or higher rate than baseline LTOT, SpO_2_ < 92%, or PaO_2_ < 60 mmHg without oxygen supplementation) or mechanical ventilation
No hospital admission in the past 28 days
Exclusion criteria
No clinical data available from the first 48 h after initial admission
Pneumonitis induced by immunosuppressive regimen
Aspiration pneumonia
Infarction pneumonia
Cardiac decompensation with pneumonia superimposed on pulmonary edema
Missing data on immunosuppressive medication at the time of admission
Documented treatment restrictions
No medical reports available

*CT* computed tomography, *LTOT* long-term oxygen therapy, *SpO*_*2*_ peripheral oxygen saturation, *PaO*_*2*_ Partial pressure of oxygen.

### Outcomes

The primary endpoint was severe pneumonia, a composite endpoint consisting of in-hospital mortality, respiratory failure requiring invasive mechanical ventilation (IMV), acute kidney injury (AKI) requiring kidney replacement therapy (KRT), and need for vasopressor therapy.

The secondary outcomes were in-hospital mortality, 28-day mortality, ICU admission, need for vasopressor therapy, IMV, high-flow nasal cannula (HFNC) or non-invasive mechanical ventilation (NIV), acute kidney injury (AKI) stage according to KDIGO or need for KRT, and persistent impairment of estimated glomerular filtration rate (eGFR) at discharge in comparison to baseline eGFR. To ensure the validity of the data on 28-day mortality, we verified that at least one follow-up visit was performed at our transplant center or by the home nephrologist more than 28 days after the initial hospital admission for patients, who did not experience in-hospital death and whose hospitalization was shorter than 28 days.

### Microbiology

Only pathogens identified within the first 7 days after admission were considered to be related to the acute CAP episode and were included in the analysis. Causative pathogens were identified as detailed in Item S3.

### Risk scores and ROC analysis

Missing values for predictor variables were either calculated based on other variables available or by performing multiple imputation (MI) as detailed in Item S4. To assess the effect of MI on the results of the subsequent analyses, complete case analysis was performed as a sensitivity analysis. The following eight risk scores were calculated for each patient’s first pneumonia, separately for each of the five imputed datasets.

*CRB-65* [10], *CURB-65* [11], *DS-CRB-65* [12], *qSOFA* [13], and *NEWS-2* [20] were calculated as described elsewhere from the first clinical data available from each patient after admission. It was ensured that no data were included after the endpoint was reached.

SOFA [16], PSI [13], and *IDSA* [14] for CAP were slightly modified to account for unavailable information as detailed in Item S5. In case of complete case analysis, the scores were calculated only for patients, for whom all necessary information was available.

### Statistical analysis

Statistical analysis was performed using *R studio 2021.09.2* with *R version 4.1.2*. as detailed in Item S6. Descriptive analysis was performed using *base R* and *R package psych* [23]. Plotting was performed using *R package ggplot2* [24]. Multiple imputation was performed using *R package mice* [25]. ROC analysis was performed using *R package pROC* [26] and pooling of performance metrics was performed using the function *pool_auc* from *R package psfmi* [27]*.*

## Results

**Fig.1:**
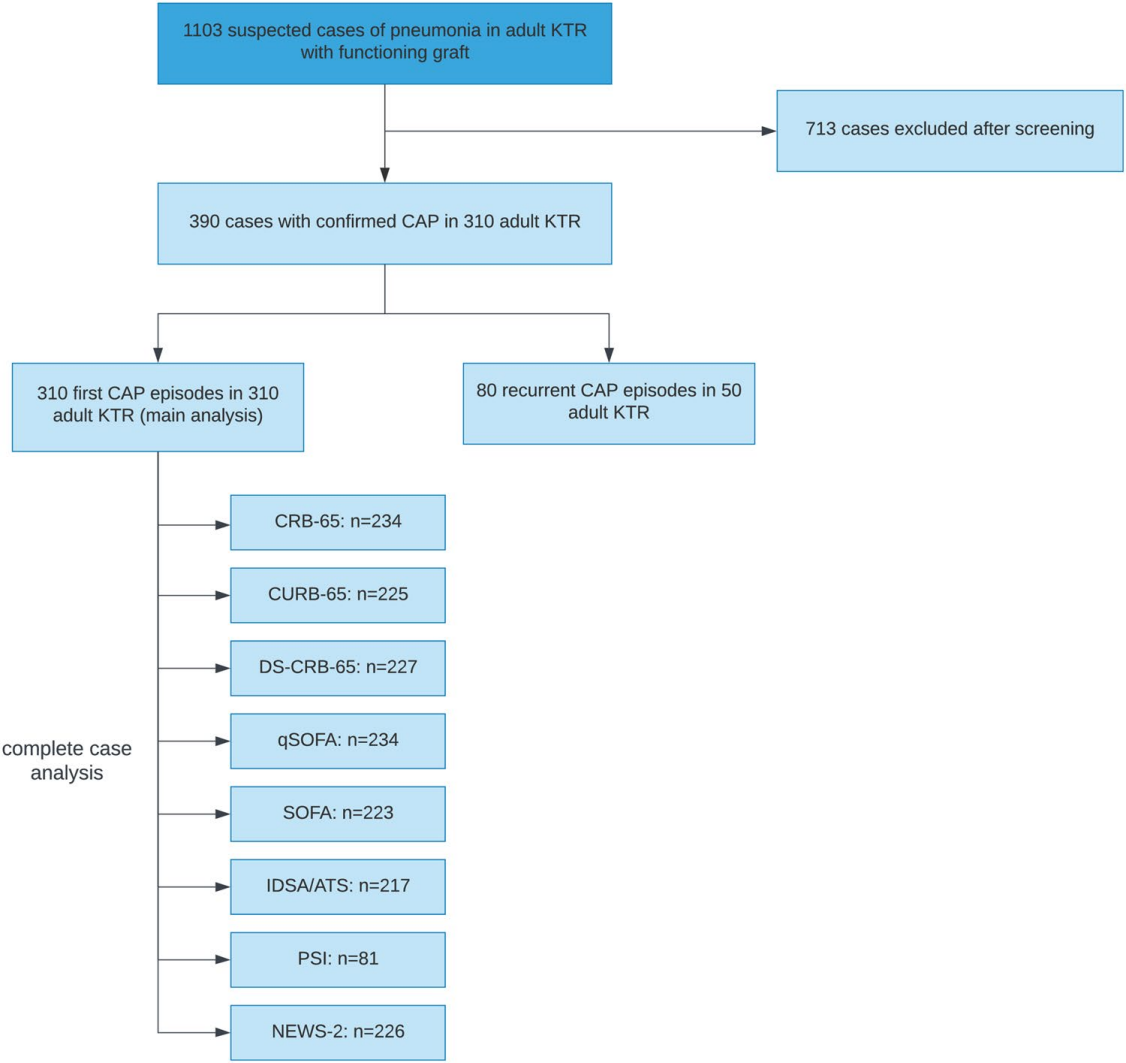
Patient flow diagram. After applying all inclusion and exclusion criteria, 390 cases of confirmed CAP in 310 adult KTR were retrieved from our database. We included the first CAP episode for each patient into the final analysis to ensure statistical independence and applied multiple imputation for missing variables necessary to calculate risk scores. For complete case analysis, risk scores were calculated only when all variables were available, resulting in different sample size depending on the risk score, KTR—kidney transplant recipients, CAP—community-acquired pneumonia

In total, 1103 KTR cases were screened. After applying all inclusion and exclusion criteria described in Table 1, 390 cases of CAP were retrieved from our database. To ensure statistical independence for each case, we included the first CAP for patients with more than one CAP episode into our main analysis, resulting in 310 cases. In 50 patients, more than one CAP episode occurred, resulting in 80 recurrent CAP episodes. The patient flow is shown in Fig. 1, and comorbidities, transplant-related characteristics, imaging as well as laboratory parameters are summarized in Table 2. The number and distribution of missing values for each risk score are shown in Figures S1–S8.

**Table 2:** Patient charcteristics

Patient characteristics	310 first CAP in 310 patients	80 recurrent CAP episodes
Demographics		
Age in years	58.7 (46.8–68.2)	60.1 (54.7–67.9)
Sex female /male	35.8% (111) / 64.2% (199)	33.8% (27) / 66.2% (55)
*Comorbidities*		
Cardiovascular risk factors		
Diabetes mellitus	31.3% (97)	45.0% (36)
Arterial hypertension	92.6% (287)	97.5% (80)
Cardiovascular disease		
Coronary artery disease	25.8% (80)	28.8% (24)
History of myocardial infarction	9.7% (30)	7.5% (6)
Peripheral artery disease	8.7% (27)	13.8% (11)
History of stroke	6.8% (21)	10.0% (8)
Pulmonary disease		
COPD	8.7% (27)	28.8% (23)
Asthma bronchiale	1.6% (5)	0% (0)
Malignancy	21.0% (65)	23.8% (20)
Liver disease	18.2% (56)	22.5% (18)
Transplantation		
Transplant age (years)	4.0 (1.5–7.4)	7.6 (3.2–11.6)
CNI-based immunosuppression	83.2% (258)	60.0% (49)
Tacrolimus	58.1% (180)	26.3% (21)
Ciclosporin	25.2% (78)	33.8% (28)
Belatacept-based immunosuppression	3.9% (12)	10.0% (8)
Steroid treatment	82.3% (255)	91.0% (73)
MPA treatment	78.4% (243)	88.8% (72)
mTOR inhibitor treatment	11.9% (37)	7.5% (6)
Baseline eGFR (ml/min/1.73m^2^)	49.8 ± 22.9	43.7 ± 25.6
Imaging		
Multilobar infiltrates	55.2% (171)	56.3% (46)
Bilateral pneumonia	51.3% (159)	47.5% (38)
Pleural effusion	22.6% (70)	32.5% (26)
Laboratory values		
CRP (mg/L)	78.3 (37.6–140.3)	59.7 (29.8–149.2)
Procalcitonin (ug/L)	0.33 (0.12–4.32)	0.20 (0.08–2.62)
WBC (/nL)	8.7 (6.1–12.5)	8.9 (6.7–13.9)
Lymphocytes (/nL)	0.49 (0.27–0.99)	0.76 (0.36–1.47)
Neutrophils(/nL)	6.1 (3.7–10.0)	6.5 (3.5–11.6)
Hemoglobin (g/dL)	11.5 ± 1.90	11.2 ± 2.1
Thrombocytes (/nL)	234 ± 99	229 ± 86
eGFR at admission (ml/min/1.73m^2^)	33.2 (20.7–49.0)	24.1 (18.3–44.0)

Baseline characteristics are provided as median (interquartile range) or mean ± standard deviation (SD) if not stated otherwise. Laboratory values are provided after pooling the values from 5 multiply imputed datasets

*COPD* chronic obstructive pulmonary disease, *CNI* calcineurin inhibitors, *MPA* mycophenolic acid, *mTOR* mammalian target of rapamycin, *CRPC*-reactive protein, *WBC* white blood cell count

### Outcomes

**Fig. 2:**
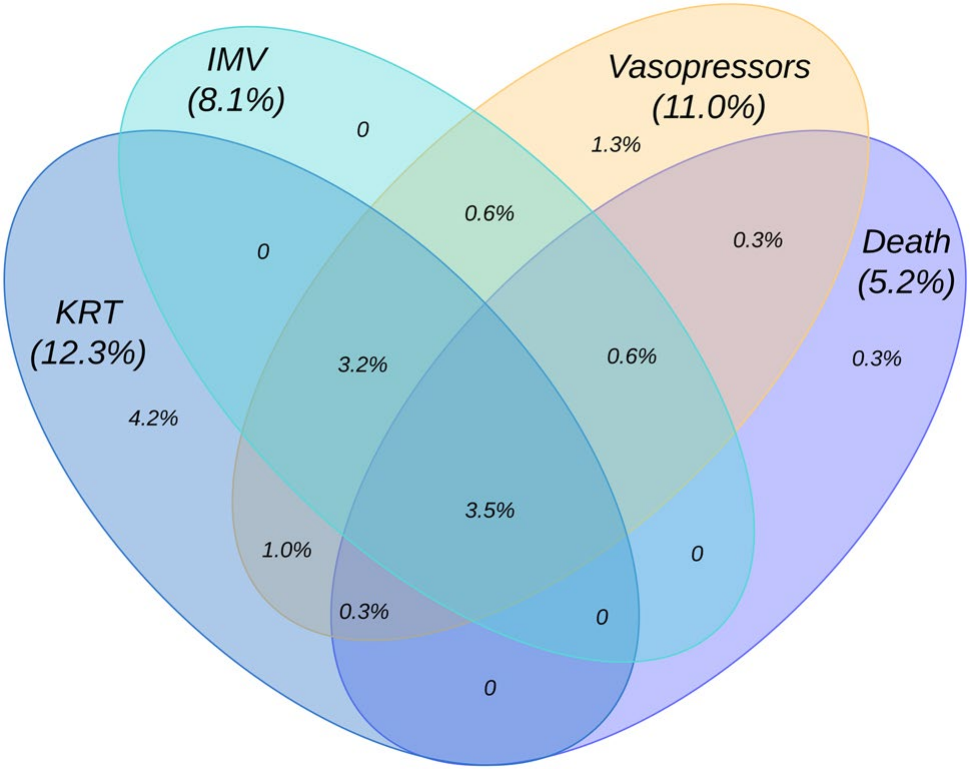
Frequencies of organ failure treatments and in-hospital death qualifying for the primary endpoint. *IMV* invasive mechanical ventilation, *KRT* kidney replacement therapy

The combined primary endpoint was reached in 48 patients (15.5%); 16 out of 310 patients (5.2%) died in hospital (Fig. 2 and Table 3).

**Table 3:** Outcomes of community-acquired pneumonia in kidney transplant recipients

Outcome	310 first pneumonias in 310 patients	First COVID *n* = 43	First Non COVID *n* = 267	*p* value Non-COVID vs. COVID	80 recurrent pneumonias in 50 patients
Primary outcome					
Severe pneumonia (IMV, KRT, vasopressor therapy, in-hospital death)	15.5% (48/310)	32.6% (14/43)	12.7% (34/267)	**0.011**	13.8% (11/80)
Mortality					
28-day mortality	3.2% (10/310)	11.6% (5/43)	1.9% (5/267)	–	5.0% (4/80)
In-hospital death	5.2% (16/310)	18.6% (8/43)	3.0% (8/267)	**0.014**	5.0% (4/80)
ICU admission	21.9% (68/310)	37.2% (16/43)	19.5% (52/267)	–	18.8% (15/80)
ICU duration	6 days (3–17 days)	10.5 days (5.75–32 days)	4.5 days (2–17 days)		6 days (3–9 days)
IMV	8.1% (25/310)	23.3% (10/43)	5.6% (15/267)	**0.011**	6.3% (5/80)
IMV duration	9.5 days (6–34 days)	13 days (5–32 days)	9 days (7–38 days)		4.5 days (3.75–6.25 days)
HFNC/NIV	10.3% (32/310)	14.0% (6/43)	9.7% (26/267)	–	11.3% (9/80)
HFNC/NIV duration	4 days (2–7 days)	4.5 days (3–6.75 days)	4 days (1.5–8 days)		3 days (1.25–5.5 days)
Vasopressor therapy	11.0% (34/310)	25.6% (11/43)	8.6% (23/267)	**0.018**	7.5% (6/80)
Start of vasopressor therapy	2 days after admission (0–6 days)	7 days after admission (2–12 days)	1 days after admission (0–4 days)		1.5 days after admission (0.25–2 days)
*Renal outcomes*					
AKI					
No AKI	27.1% (84/310)	20.9% (9/43)	28.1% (75/267)	–	26.3% (21/80)
KDIGO 1	49.7% (154/310)	51.2% (22/43)	49.4% (132/267)	–	41.3% (33/80)
KDIGO 2	5.5% (17/310)	11.6% (5/43)	4.5% (12/267)	–	5.0% (4/80)
KDIGO 3	17.7% (55/310)	16.3% (7/43)	18.0% (48/267)	–	27.5% (22/80)
RRT	12.3% (38/310)	23.3% (10/43)	10.5% (28/267)	0.066	12.5% (10/80)
Persistent renal impairment at discharge					
eGFR at baseline at discharge	77.1% (239/310)	81.4% (35/43)	76.4% (204/267)	–	78.8% (63/80)
eGFR loss of 5–10 ml/min/1.73m^2^	11.0% (34/310)	2.3% (1/43)	12.4% (33/267)	–	10.0% (8/80)
eGFR loss of 10–20 ml/min/1.73m^2^	8.7% (27/310)	9.3% (4/43)	8.6% (23/267)	–	7.5% (6/80)
eGFR loss > 20 ml/min/1.73m^2^	3.2% (10/310)	7.0% (3/43)	2.6% (7/267)	–	3.8% (3/80)
In-hospital graft loss	1.9% (6/310)	0% (0/43)	2.2% (6/267)	–	2.5% (2/80)

Disease severity between COVID- and Non-COVID-pneumonia was compared for the primary outcome and each single outcome it includes—in-hospital death, need for dialysis, vasopressor therapy or invasive mechanical ventilation

*IMV* invasive mechanical ventilation, *KRT* kidney replacement therapy, *ICU* intensive care unit, *HFNC* high-flow nasal canula, *NIV* non-invasive ventilation, *AKI* acute kidney injury

Among the 310 patients, 43 patients had COVID-19. Patients with COVID-19 developed severe pneumonia more frequently (32.6%; 14/43) than non-COVID-19 patients (12.7%; 34/267; *p* = 0.011) and had higher rates of secondary endpoints. In-hospital mortality was 18.6% (8/43) vs. 3.0% (8/267; *p* = 0.014), IMV was performed in 23.3% (10/43) vs. 5.6% (15/267; *p* = 0.011), vasopressor treatment in 25.6% (11/43) vs. 8.6% (23/267; *p* = 0.018), and KRT in 23.3% (10/46) vs. 10.5% (28/267, *p* = 0.066) (Table 3) in COVID-19 vs. non-COVID-19 patients, respectively.

With respect to renal outcomes, 27.1% (84/310) of all patients had no AKI, 49.7% (154/310) stage 1 AKI, 5.5% (17/310) stage 2 AKI, 17.7% (55/310) stage 3 AKI, and 12.3% (38/310) required KRT during the admission. We further analyzed which proportion of patients had persistent impairment of eGFR at discharge. While eGFR was completely restored in the majority of patients (77.1%—239/310), 11.0% (34/310) had eGFR loss of 5–10 ml/min/1.73 m^2^, 8.7% (27/310) eGFR loss of 10–20 ml/min/1.73 m^2^, 3.2% (10/310) eGFR loss of > 20 ml/min/1.73m^2^, and 1.9% (6/323) lost their graft function in-hospital, meaning permanent return to dialysis.

### Pathogens

**Fig. 3:**
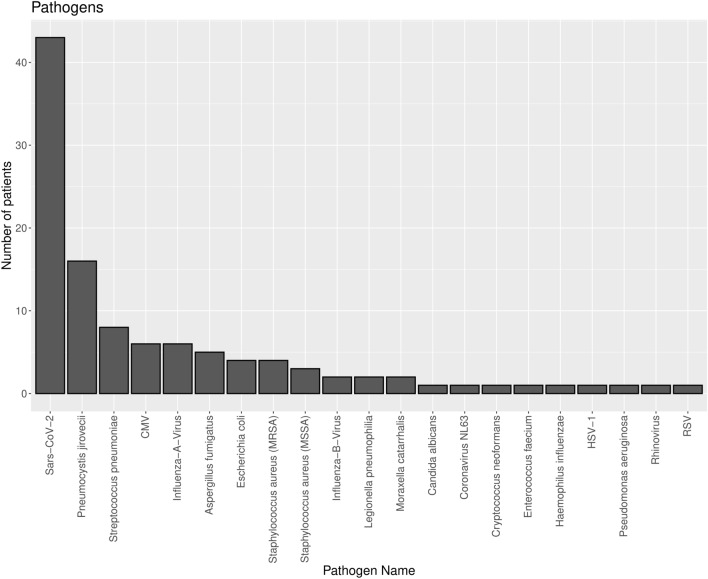
Distribution of causative pathogens for the first episode of community-acquired pneumonia in kidney transplant recipients. *SARS-CoV-2* severe acute respiratory syndrome coronavirus 2, *CMV* cytomegalovirus, *MRSA* methicillin-resistant staphylococcus aureus, *MSSA* methicillin-susceptible staphylococcus aureus, *HSV* herpes simplex virus, *RSV* respiratory syncytial virus

In 64.5% (200/310) of cases, no causative pathogen was identified. In the remaining 110 cases, SARS-CoV-2 (39.0%), Pneumocystis jirovecii (PjP; 14.5%), Streptococcus pneumoniae (7.3%), CMV (5.5%), and influenza A (5.5%) were the most frequent ones (Fig. 3 and Table 4). In the first year after transplantation, PjP and CMV were more common than in the following years (Figures S9 and S10), with discontinuation of prophylactic treatment often preceding infection (Table S1). Recurrent pneumonias show comparable results for causative pathogens with the exception that pneumocystis is not found as causative pathogen in recurrent pneumonia (Figure S11). A more detailed analysis of CMV and PjP pneumonia is shown in Table S1 and Item S7.

**Table 4:** Causative pathogens isolated in kidney transplant recipients presenting with community-acquired pneumonia

Main pathogen	First pneumonia	Recurrent pneumonias
No. pathogen isolated	200	45
Causative pathogen isolated	110	35
Viral	55.5% (61)	17 (48.6%)
Bacterial	23.6% (26)	18 (51.4%)
Fungal	20.9% (23)	–
Sars-CoV-2	39.0% (43)	25.7% (9)
*Pneumocystis jirovecii*	14.5% (16)	–
*Streptococcus pneumoniae*	7.3% (8)	8.6% (3)
CMV	5.5% (6)	8.6% (3)
Influenza-A-Virus	5.5% (6)	2.9% (1)
*Aspergillus fumigatus*	4.5% (5)	–
*Escherichia coli*	3.6% (4)	2.9% (1)
*Staphylococcus aureus* (MRSA)	3.4% (4)	–
*Staphylococcus aureus* (MSSA)	2.7% (3)	5.7% (2)
Influenza-B-Virus	1.8% (2)	2.9% (1)
*Legionella pneumophilia*	1.8% (2)	–
*Moraxella catarrhalis*	1.8% (2)	–
*Haemophilus influenzae*	0.9% (1)	8.3% (3)
HSV-1	0.9% (1)	2.9% (1)
*Pseudomonas aeruginosa*	0.9% (1)	5.7% (2)
*Candida albicans*	0.9% (1)	–
Coronavirus NL63	0.9% (1)	–
*Cryptococcus neoformans*	0.9% (1)	–
*Enterococcus faecium*	0.9% (1)	–
Rhinovirus	0.9% (1)	–
RSV	0.9% (1)	–
*Enterobacter aerogenes*	–	2.9% (1)
*Stenotrophomonas maltophilia*	–	2.9% (1)
VZV	–	2.9% (1)
Acinetobacter species	–	2.9% (1)
*Klebsiella oxytoca*	–	2.9% (1)
*Klebsiella pneumoniae*	–	2.9% (1)
*Metapneumovirus*	–	2.9% (1)
*Mycobacterium kansasii*	–	2.9% (1)
*Proteus mirabilis*	–	2.9% (1)

*SARS-CoV-2* severe acute respiratory syndrome coronavirus 2, *CMV* cytomegalovirus, *MRSA* methicillin-resistant staphylococcus aureus, *MSSA* methicillin-susceptible staphy

lococcus aureus, *HSV* herpes simplex virus, *RSV* respiratory syncytial virus, *VZV* varizella zoster virus

### Risk scores

Next, we validated eight different risk scores for pneumonia, sepsis, or general risk assessment, namely CRB-65, CURB-65, DS-CRB-65, qSOFA, SOFA, PSI, IDSA/ATS minor criteria, and NEWS-2. For every score, prediction of in-hospital mortality as well as severe pneumonia were assessed by ROC analysis. Instead of using the previously described cutoffs, we assessed threshold-dependent metrics such as sensitivity, specificity, positive predictive value (PPV), and negative predictive value (NPV) based on the Youden index.

**Fig. 4:**
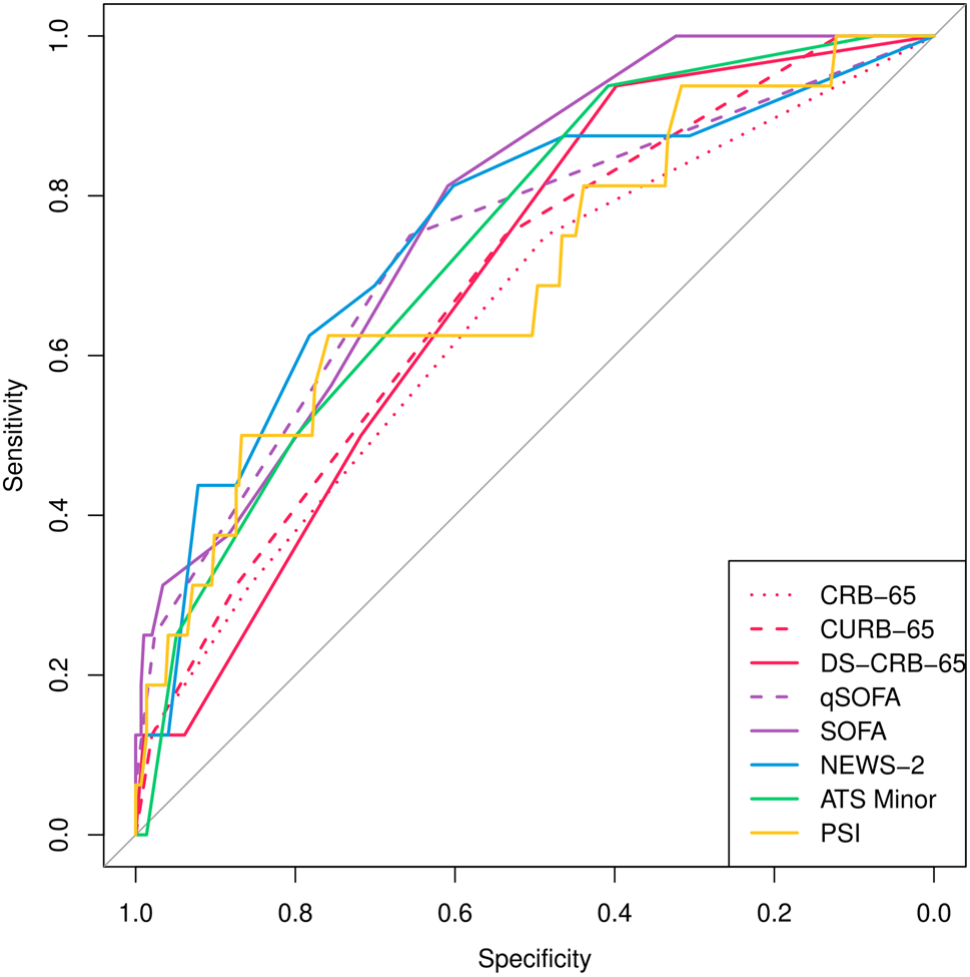
ROC analysis of eight different risk scores for prediction of in-hospital mortality in 310 kidney transplant recipients with community-acquired pneumonia. Variables for the risk scores were assessed up to 48 h after hospital admission, but always before an endpoint occurred. Missing values were imputed performing multiple imputation and the ROC curves for one out of five multiply imputed datasets is shown here

For prediction of death, all risk scores achieved a NPV of at least 0.97 with the cutoffs shown in Table 5. SOFA and NEWS-2 showed the highest AUC, with 0.794 (0.679–0.875) and 0.741 (0.574–0.858), respectively (Table 5 and Fig. 4).

**Table 5:** Predictive performance of eight different risk scores for prediction of in-hospital mortality in kidney transplant recipients hospitalized for community-acquired pneumonia

Score	AUC	Sens	Spec	PPV	NPV	Youden index	Cutoff	pSOFA	pNEWS-2
CRB-65	0.640 (0.471–0.780)	0.680 (0.196–0.949)	0.564 (0.114–0.929)	0.083 (0.007–0.529)	0.970 (0.937–0.986)	1.264	0.5	0.128	0.271
CURB-65	0.676 (0.516–0.803)	0.738 (0.268–0.956)	0.528 (0.189–0.843)	0.086 (0.020–0.297)	0.974 (0.940–0.989)	1.307	1.5	0.226	0.462
DS-CRB-65	0.682 (0.534–0.800)	0.938 (0.098–1.000)	0.390 (0.171–0.666)	0.079 (0.011–0.406)	0.991 (0.938–0.999)	1.352	1.5	0.114	0.363
qSOFA	0.718 (0.553–0.840)	0.727 (0.302–0.943)	0.625 (0.246–0.894)	0.105 (0.012–0.530)	0.977 (0.944–0.991)	1.410	0.5	0.297	0.708
SOFA	0.794 (0.679–0.875)	0.813 (0.436–0.961)	0.638 (0.375–0.838)	0.114 (0.018–0.468)	0.985 (0.959–0.995)	1.466	2.5	–	0.459
PSI	0.696 (0.519–0.829)	0.638 (0.300–0.879)	0.812 (0.380–0.968)	0.150 (0.057–0.339)	0.976 (0.947–0.989)	1.418	136.5	0.225	0.621
IDSA/ATS-Minor	0.733 (0.586–0.842)	0.881 (0.207–0.995)	0.430 (0.181–0.720)	0.085 (0.019–0.310)	0.987 (0.937–0.997)	1.379	1.5	0.415	0.772
NEWS-2	0.741 (0.574–0.858)	0.715 (0.347–0.922)	0.774 (0.297–0.965)	0.141 (0.047–0.352)	0.981 (0.949–0.993)	1.460	5.5	0.459	–

*P* values are the median *p* values assessed by comparing the ROC curve against the ROC curve for SOFA or NEWS-2 using the method by DeLong in 5 multiply imputed datasets

*AUC* area under the curve of the receiver operating characteristic, *Sens* sensitivity, *Spec* specificity, *PPV* positive predictive value, *NPV* negative predictive value

**Fig. 5:**
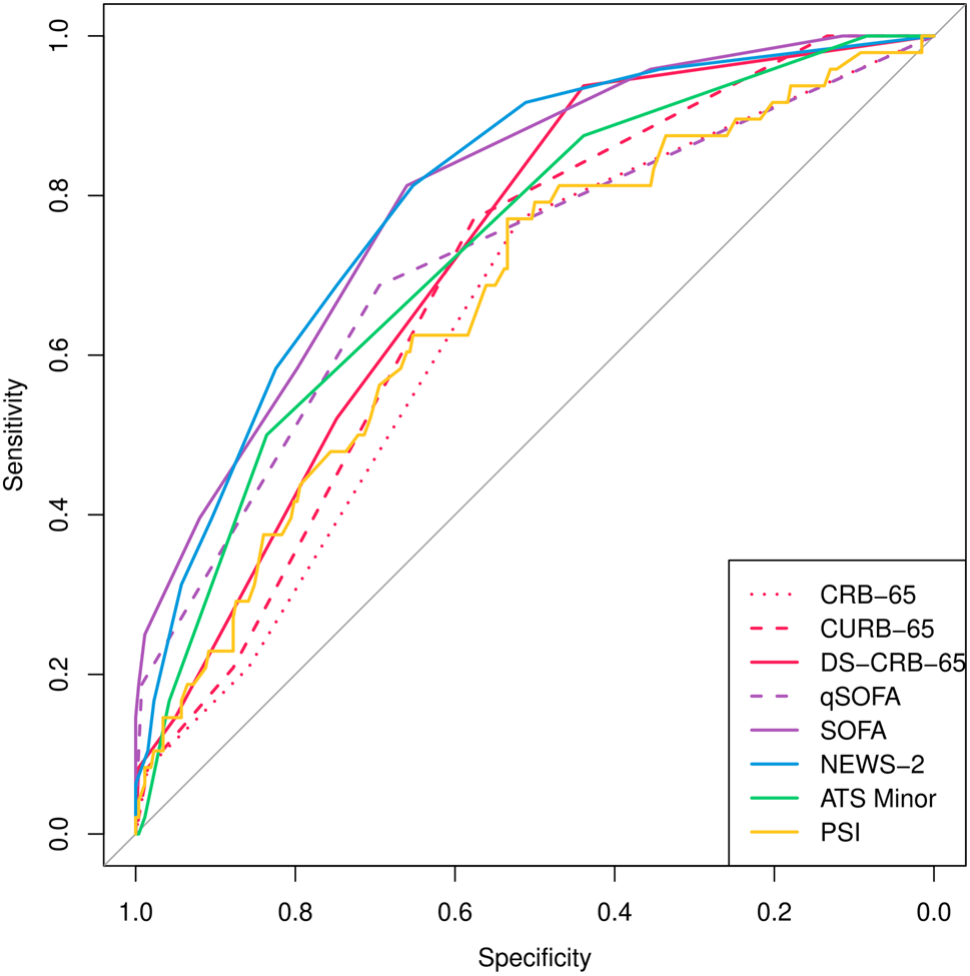
ROC analysis of eight different risk scores for predicting the occurrence of the primary endpoint. The composite endpoint of invasive mechanical ventilation, vasopressor treatment, dialysis, or in-hospital mortality in 310 kidney transplant recipients with community-acquired pneumonia was assessed up to 48 h after hospital admission, but always before an endpoint occurred. Missing values were imputed performing multiple imputation and the ROC analysis for one out of five multiply imputed datasets is shown here

For prediction of severe pneumonia, SOFA and NEWS-2 achieved the highest AUC, with 0.823 (0.747–0.880) and 0.784 (0.691–0.855), respectively (Table 6 and Fig. 5). Notably, SOFA showed a significantly higher AUC than all other risk scores. The only exception was NEWS-2, which showed significantly higher AUC than all other risk scores except for SOFA and IDSA/ATS minor criteria.

**Table 6:** Predictive performance of eight different risk scores for prediction of severe pneumonia in kidney transplant recipients hospitalized for community-acquired pneumonia

Score	AUC	Sens	Spec	PPV	NPV	Youden index	Cutoff	pSOFA	pNEWS-2
CRB-65	0.633 (0.526–0.728)	0.771 (0.562–0.899)	0.493 (0.302–0.687)	0.219 (0.167–0.282)	0.922 (0.871–0.954)	1.295	0.5	** < 0.001**	** < 0.001**
CURB-65	0.675 (0.572–0.764)	0.771 (0.620–0.875)	0.546 (0.359–0.722)	0.239 (0.181–0.308)	0.929 (0.883–0.958)	1.345	1.5	**0.003**	**0.011**
DS-CRB-65	0.714 (0.618–0.795)	0.940 (0.234–0.999)	0.423 (0.279–0.582)	0.231 (0.169–0.308)	0.974 (0.846–0.996)	1.380	1.5	**0.017**	**0.0496**
qSOFA	0.702 (0.593–0.792)	0.709 (0.432–0.886)	0.641 (0.320–0.872)	0.269 (0.116–0.509)	0.923 (0.877–0.952)	1.382	0.5	**0.020**	**0.003**
SOFA	0.823 (0.747–0.880)	0.829 (0.631–0.933)	0.692 (0.571–0.791)	0.331 (0.245–0.430)	0.956 (0.914–0.978)	1.528	2.5	–	0.402
PSI	0.666 (0.397–0.888)	0.695 (0.397–0.888)	0.623 (0.329–0.848)	0.258 (0.162–0.383)	0.918 (0.866–0.951)	1.328	109.5	** < 0.001**	**0.012**
ATS-Minor	0.730 (0.639–0.805)	0.806 (0.279–0.978)	0.550 (0.121–0.916)	0.260 (0.137–0.437)	0.935 (0.858–0.972)	1.352	1.5	**0.026**	0.170
NEWS-2	0.784 (0.691–0.855)	0.809 (0.532–0.940)	0.659 (0.481–0.801)	0.301 (0.197–0.430)	0.948 (0.894–0.975)	1.469	3.5	0.402	–

*P* values are the median p values assessed by comparing the ROC curve against the ROC curve for SOFA or NEWS-2 using the method by DeLong in 5 multiply imputed datasets

*AUC* area under the curve of the receiver operating characteristic, *Sens* sensitivity, *Spec* specificity, *PPV* positive predictive value, *NPV* negative predictive value

**Fig. 6:**
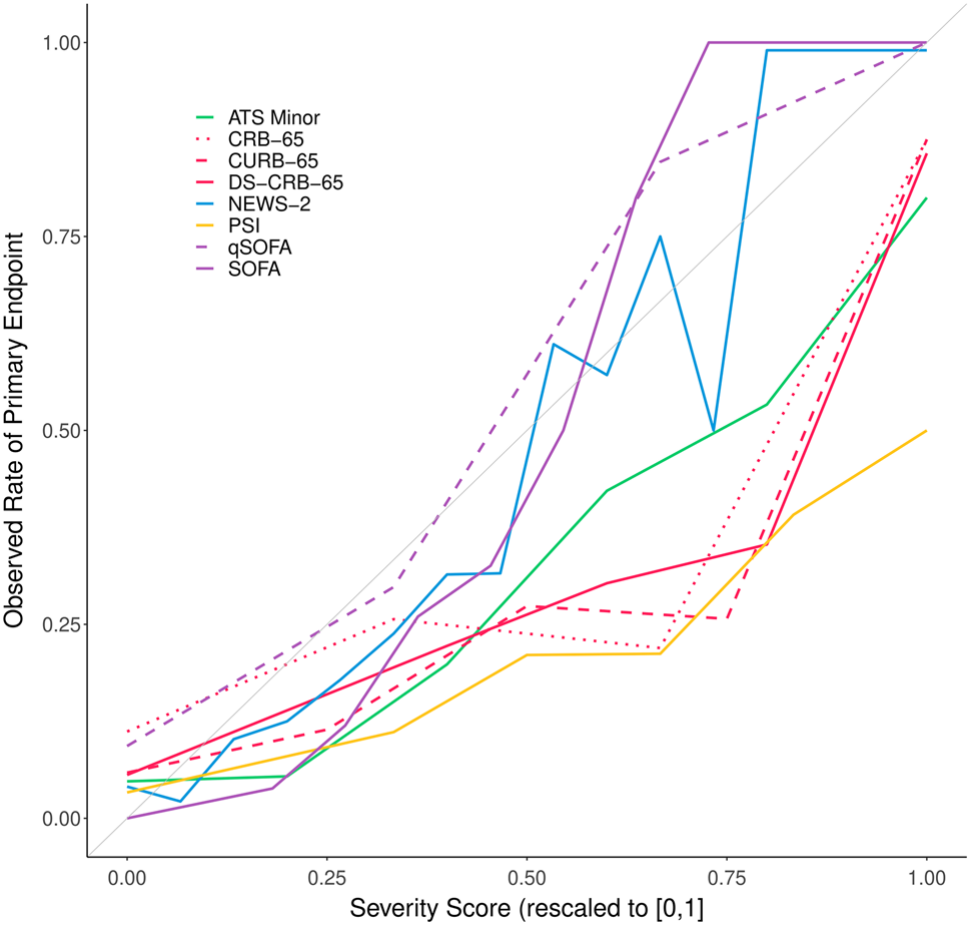
Percentage of patients with primary endpoint (severe pneumonia) in dependence on risk scores. Scores were rescaled to the unit interval for this purpose. We pooled scores in order to deal with sparsely filled score classes as follows: for DS-CRB-65 ≤ 1, for SOFA ≤ 1 and ≥ 10, for ATS-Minor ≥ 5, for NEWS-2 10–13, and 14–15, and for PSI < 60 and ≥ 180. Missing values were imputed performing multiple imputation and the data from one out of five multiply imputed datasets are shown here

The observed rate of severe pneumonia and in-hospital death for each value of each score are shown in Table S2. To summarize those results, all risk scores were rescaled to [0,1] and plotted against the observed rate of severe pneumonia for each observed score value in Fig. 6. qSOFA, SOFA, and NEWS-2 all run closely to the diagonal, suggesting good calibration. This means that higher score values indicate higher event rate, while the lowest score values indicate an event rate close to 0% and the highest score values indicate event rates close to 100%. The other risk scores slightly overestimate the risk of severe pneumonia for higher score values, but in general show an increase in event rate with higher score values as well.

We separately analyzed the correlation of each variable included into the risk scores with the primary outcome. In the univariable analysis, we found 15 out of 27 variables to be significantly correlated with the primary outcome: altered mental status, heart rate, respiratory rate, SpO_2_, need for oxygen supplementation, Horovitz index, sodium, blood glucose, BUN, creatinine, bilirubin, congestive heart failure, pleural effusion, and multilobular infiltrates (Table S3).

### Sensitivity analyses

To verify that computational decisions do not affect the results and conclusions, the following sensitivity analyses were performed.

For all scores, comparable or slightly better results were achieved when no multiple imputation, but complete case analysis was performed as shown in Tables S4/S5.

Since patients with COVID-19 were included when meeting the CAP definition above, we analyzed the predictive performance of all eight risk scores separately for COVID vs. Non-COVID patients. We found that SOFA and NEWS-2 still performed best in detecting the primary endpoint with AUC of 0.804 (0.648–0.901) and 0.787 (0.614–0.895) for COVID-19 pneumonia, and 0.843 (0.749–0.906) and 0.790 (0.684–0.867) for non-COVID-19 pneumonia, respectively (Table S6).

## Discussion

In the present study, we validated a comprehensive set of eight different risk scores established to describe disease severity or predict outcomes in pneumonia and sepsis in a large cohort of KTR hospitalized for CAP. We compared the discriminative power with respect to in-hospital mortality and severe pneumonia.

We included risk scores developed to predict CAP outcomes such as CRB-65, CURB-65, DS-CRB-65, PSI, and IDSA/ATS minor criteria, and added SOFA, qSOFA and NEWS-2. Although SOFA as well as its simplified version qSOFA were initially defined to describe the sequence of complications in distinct organs in sepsis, they have been widely used to predict mortality and severe disease course in CAP [17, 18, 28]. Similarly, early warning scores such as NEWS have been investigated as risk stratification tools for CAP, with NEWS-2 being extensively studied within patients with COVID-pneumonia in the last years [29–32].

ROC analysis revealed that SOFA and NEWS-2 discriminate KTR at risk for severe pneumonia significantly better than the other risk scores investigated. This finding can be explained by three clinically important observations:

First, in contrast to immunocompetent patients [17, 28, 33], age did not significantly correlate with severe pneumonia in our analysis. Accordingly, risk scores containing an age criterion, such as CRB-65, predicted the occurrence of severe pneumonia with less accuracy than risk scores containing similar amounts of variables without an age criterion, such as qSOFA. In comparison to immunocompetent patients with CAP in Germany, the median age of our study population was considerably lower (59 vs. 76 years) [33], which may further explain why age-dependent scores like CRB-65 perform worse in our cohort. This is in line with a secondary analysis from a recent international multi-center study, where immunocompromised patients were significantly younger than immunocompetent patients with CAP [34]. As described before, immunosuppression might facilitate the development of severe pneumonia courses with poor prognosis at a younger age [21, 35]. Therefore, risk scores not relying on age might be preferable in immunocompromised patients with pneumonia [19], which might be applicable for KTR hospitalized for CAP as well.

Second, scores including granular information on pulmonary changes (IDSA/ATS-Minor criteria, PSI, NEWS-2, SOFA) as well as extrapulmonary organ failure (SOFA) performed better, except for PSI. The latter may be explained by extensive imputation, which was necessary for PSI. It is not surprising that SOFA performed best in identifying severe pneumonia, since increases in creatinine and decreases in mean arterial pressure and Horovitz index regularly precede each single outcome composing the primary endpoint.

Third, NEWS-2 showed comparable discrimination as SOFA by only including clinical variables. A possible explanation is that NEWS-2 assesses clinical variables such as respiratory rate, temperature, and blood pressure with the greatest granularity. This might be relevant, since clinical characteristics of immunocompromised patients may differ from immunocompetent patients with CAP [35]. For example, temperature changes indicating systemic infection may be less pronounced in KTR with CAP due to immunosuppression. As opposed to PSI or IDSA/ATS-Minor criteria detecting only large temperature deviations, NEWS-2 might be better suited in this cohort.

To date, only a few studies have investigated risk scores in immunocompromised patients with CAP. In line with our results, other authors found a moderate prognostic value of CRB-65 and qSOFA in immunocompromised patients with pneumonia. Frantz et al. found similar AUC of 0.630 for CRB-65 and 0.688 for qSOFA in the prediction of severe CAP in a cohort of 198 immunocompromised patients, including 18 KTR [19]. Carrabba et al. found a lower AUC of 0.57 for CRB-65 than in our study, but comparable AUC of 0.68 for PSI and 0.62 for CURB-65 when predicting mortality in immunocompromised patients with pneumonia [21].

Reduced performance of qSOFA compared to SOFA has also been shown in the large German cohort PROGRESS predicting severe CAP in immunocompetent patients with CAP [17]. In line with our results, improvement of CRB-65 by adding oxygenation and comorbidities (DS-CRB-65) was observed in the CAPNETZ cohort of immunocompetent German CAP patients [37].

While AUC is a suitable summary statistic with respect to discrimination, practical benefit of risk scores depends on threshold- and incidence-dependent metrics such as PPV and NPV. Standard pneumonia scores like CRB-65 have initially been developed to predict mortality. The threshold of CRB-65 ≥ 1 to indicate hospital admission is chosen to achieve high NPV, so that patients with the lowest score have a very low risk of mortality and can in most cases receive outpatient treatment.

In our analysis, all investigated scores had NPV for in-hospital mortality above 97%. This is due to a low mortality rate of 5.2% and does not suffice to identify patients with low mortality risk for most scores. Only zero points in CURB-65, SOFA, or IDSA/ATS-Minor criteria indicated 0% risk of mortality in our cohort. Correspondingly, only zero points in CURB-65 or IDSA/ATS-Minor criteria indicated 0% risk for development of severe pneumonia. One could argue that for patients with zero points in CURB-65 or IDSA/ATS-Minor criteria, outpatient treatment could be considered due to a very low risk of severe disease. For the other scores, even patients with low score values develop severe pneumonia in a relevant proportion and should be hospitalized and closely monitored.

Furthermore, all the scores were calibrated with respect to the primary endpoint. This means that for the lowest score values, an event rate close to 0% was observed, and for higher score values, a proportionally higher event rate was observed. The latter was most pronounced for SOFA, NEWS-2, and qSOFA, for which the highest score values indicated a 100% risk of severe pneumonia. The other scores overestimate the risk of severe pneumonia to a different extent.

Previous research on risk scores for CAP in immunocompromised patients was mostly performed in heterogeneous groups in terms of underlying disease, treatment regimen, and severity of immunosuppression. Moreover, controversy remains regarding the conditions to be included in the definition of immunocompromised patients with CAP [5]. Hence, studying CAP in a distinct cohort of KTR can lead to more reliable results than studying patients receiving different types of immunosuppressive therapy.

To our knowledge, this is the first study to comprehensively assess the prognostic value of risk scores in a large cohort of KTR hospitalized for CAP.

## Limitations

Generally, due to the retrospective and single-center design, the results of this study remain explorative and hypothesizing and need replication within prospective multi-center cohorts.

While many risk scores for CAP have been developed to predict mortality, we chose severe CAP as our primary endpoint for two reasons: (1) due to the small number of deaths, the estimation of discrimination would be less reliable for death as primary endpoint, (2) every item of our composite endpoint is of high importance for KTR with CAP, and intensified management strategies have been shown to improve clinical outcomes in immunocompetent patients [38], as well as in immunocompromised patients with CAP, who are critically ill [39, 40].

Consequently, the exact values for AUC and threshold-dependent metrics, such as sensitivity and specificity, are less reliable for in-hospital death than for the primary endpoint.

Owing to missing values for all risk scores, we used multiple imputations to perform a direct comparison of all risk scores. While sensitivity analysis showed that complete case analysis yielded comparable results in general, this was not true for PSI, where imputation was necessary for most patients. This is due to missing pH values in a large proportion of patients. Hence, PSI results must be interpreted carefully.

To reduce the amount of missing data, we used the earliest value available for each variable from the first 48 h after hospital admission for prediction. We ensured that no data after the occurrence of any endpoint of interest were included into the prediction to balance data recovery and the validity of our results.

In contrast to a recently published study [41], identification of causal pathogens was not the primary objective. The low overall detection rate of 36% might be partially explained by the retrospective study design and lack of a systematic approach to microbiological sampling.

In line with recent study results in KTR with CAP [41], we found a surprisingly low mortality rate, which might be further explained by the exclusion of patients with documented treatment restrictions in our study.

As described above, patients with COVID-pneumonia had a more severe disease course than those with non-COVID-pneumonia with respect to all relevant outcome measures, which has been described in several cohorts of KTR. Since SARS-CoV-2 will probably continue to be an important cause of CAP, especially in immunosuppressed patients, we included COVID-pneumonia in our analysis. Subgroup analysis showed differences in the AUC for most scores when used for COVID vs. non-COVID CAP. Nevertheless, NEWS-2 and SOFA-Score showed superior discrimination in predicting severe CAP for both COVID and non-COVID CAP.

## Conclusion

SOFA and NEWS-2, assessed in the first 48 h after hospital admission due to CAP, are best suited to identify KTR at risk for the development of severe CAP. CRB-65 should not be used to guide outpatient treatment in KTR, since there is a 7% risk for the development of severe pneumonia even in patients with a score of zero.

### Supplementary Information

Below is the link to the electronic supplementary material.

Supplementary file1 (DOCX 210 kb)
