# Supplementary material for: Validation of risk scores for prediction of severe pneumonia in kidney transplant recipients hospitalized with community-acquired pneumonia
Source: Infection. 2023 Nov 20;52(2):447–59. doi: 10.1007/s15010-023-02101-z (PMC10954831; doi:10.1007/s15010-023-02101-z)
Supplement: Supplementary file 1 — Supplementary file1 (DOCX 210 kb) [file 15010_2023_2101_MOESM1_ESM.docx]

**Supplementary Data**

**Supplementary Methods**

**Item S1 ICD-10 codes for pneumonia used in database query**

Specifically, we searched for a diagnosis containing the text “pneumonia” or one of the following ICD-10 codes: A21.2, A31.0, A40.3, A42.0, A37, A43, A48.1, B01.2, B25.0, B34.9, B37.1, B44.0, B44.1, B45.0, B46.0, B48.5, B59, B77.8, J10-18, J85.1, U69.01, U69.03.

**Item S2 Demographic and clinical data retrieved from the database**

Demographic information included patient sex and age at the time of hospital admission.

The clinical information we assessed comprised the first respiratory rate, heart rate, systolic and diastolic blood pressure (BP), temperature, oxygen saturation (SpO_2_), oxygen flow rate (if applicable), and mental status (altered/not altered) available in the records for each case.

We assessed the following comorbidities by reviewing the patient's medical records - (1) cardiovascular risk factors such as arterial hypertension, diabetes mellitus, and cardiovascular end-organ damage including coronary heart disease, previous myocardial infarction, congestive heart failure, peripheral artery disease, history of stroke, cerebrovascular disease, (2) active malignancy, (3) liver disease including history of hepatitis, or presence of liver cirrhosis, and its complications portal hypertension, or bleeding from esophageal varices, and (4) lung disease such as chronic obstructive pulmonary disease (COPD) and asthma bronchiale.

From our transplant database, we included the following transplantation-related information:

transplant age, immunosuppressive regimen at the time of transplantation, CMV-risk status, and baseline eGFR.

Laboratory parameters assessed included pH, blood urea nitrogen (BUN), sodium, creatinine, blood glucose, procalcitonin, C-reactive protein (CRP), white blood cell count (WBC), hemoglobin count, lymphocyte count, hematocrit. We included the first value for each parameter after hospital admission into our analysis.

**Item S3 Details of microbiological assessment**

Only pathogens identified within the first 7 days after admission were considered to be related to the acute CAP episode and were included in the analysis. Causative pathogens were identified as described by *Krüger et al.* [1] and adapted as required.

Representative sputum samples from the lower respiratory tract were defined by the criteria > 25 granulocytes and < 10 epithelial cells per low power field. Pathogens were only considered causal if at least one of the following criteria was met: 1) blood cultures yielding a bacterial or fungal pathogen (in the absence of an apparent extrapulmonary focus); 2) tracheobronchial secretions: at least ++ growth of one of the species defined as pathogens or > 10^5 cfu/ml; 3) a valid sputum sample (leukocytes 25 per 10× field) yielded one or more predominant bacterial pathogens or +++ growth. The following species were regarded as potential pathogens: Streptococcus pneumoniae, Haemophilus influenzae, Klebsiella pneumoniae, Escherichia coli and other enterobacterial species, Pseudomonas aeruginosa, Moraxella catarrhalis, Stenotrophomonas maltophilia; 4) Legionella spp.: bacterial growth in respiratory secretions or detection of urinary antigen or detection of legionella specific DNA by PCR; 5) positive urinary antigen for Streptococcus pneumoniae; 6) PCR positive for influenza virus A or B, Sars-CoV-2, RS-Virus from nasal swab or BAL; 7) PCR positive for HSV-1, rhinovirus, VZV, coronavirus NL63, Metapneumovirus from BAL; 8) Aspergillus species. were regarded as causal in the presence of positive PCR or antigen from BAL and a decision for treatment; 9) Cryptococcus neoformans was regarded as causal in presence of positive antigen in blood. 10) CMV pneumonia was defined as the detection of CMV by viral isolation, rapid culture of BAL fluid, or the detection of CMV DNA in BAL fluid combined with clinical symptoms and decision to treat with CMV-directed therapy. For patients without BAL, new CMV viremia at the time of admission, combined with clinical symptoms, typical imaging findings and decision to treat with CMV-directed therapy was regarded sufficient to justify a diagnosis of CMV pneumonia as well. For PCR-quantification, assays values over 3000 copies/ml were regarded as positive. 11) Pneumocystis jirovecii (PjP) pneumonia was regarded as causal if all of the following criteria were met: typical interstitial pulmonary infiltrates in chest X-ray or computed tomography (CT), detection of a positive immunofluorescence testing or a strongly positive PCR result (semi-quantitative) from bronchoalveolar fluid and decision to treat with PjP-directed therapy (trimethoprim/sulfamethoxazole).

**Item S4 Missing values and imputation method**

Some values like mean arterial pressure (MAP), oxygen partial pressure (PaO_2_), fraction of inspired oxygen (FiO_2_), Horovitz index, and eGFR were not always directly available, but were necessary for the following analyses. In case they were missing, we calculated them from the available variables according to the following formulas:

*MAP = diastolic BP + ⅓ (systolic BP - diastolic BP)*

*FiO_2_ (in %) = 21 + 4 * oxygen flow rate*

*PaO_2_ = 1.19*SpO_2_ – 76.19*FiO_2_* [2].

*Horovitz index = PaO_2_ / FiO_2_*

*eGFR was calculated according to the CKD-EPI 2021 formula* [3]*.*

For remaining missing values, we performed multiple imputation (MI) by chained equations using the R package *mice*[4] *(with m=5, maxit=50, method = “pmm”)*, yielding 5 complete datasets, for which the following analyses were carried out, and the results of which were reported after pooling. We chose predictive mean matching (pmm) as imputation method to ensure that no implausible data will be imputed and to enable imputation also for variables that are in fact logarithmic transformations of an underlying biological variable (e.g. pH).

**Item S5 Modifications of risk scores to account for unavailable information**

*SOFA* (sequential organ failure assessment) was slightly modified since Glascow Coma Scale (GCS) was unavailable from the medical records. Instead, every patient with altered mental status was given 1 point.

*PSI (Pneumonia severity index)* was calculated as described, with the exception that all patients were assumed not to be nursing home residents, since this information was inconsistently available in the medical reports, but only a very small proportion of our kidney transplant cohort are nursing home residents.

*IDSA (Infectious Diseases Society of America) / ATS (American Thoracic Society) minor criteria* for CAP were modified so that hypotension requiring aggressive resuscitation was replaced by hypotension defined as systolic BP < 90mmHg. Regarding the remaining criteria, we followed the IDSA/ATS guideline definitions: respiratory rate ≥ 30/min, Horovitz index ≤ 250 mmHg, BUN ≥ 20 mg/dL as uremia, leukocyte count < 4/nL as leukopenia, thrombocyte count < 100/nL as thrombocytopenia, and hypothermia as temperature < 36°C.

**Item S6 Detailed statistical analysis plan**

To assess the predictive performance of each risk score for the primary outcome and in-hospital death, we performed receiver operating characteristics (ROC) analysis using the R package *pROC* [5].

Estimates of area under curve (AUC) and the respective 95% confidence intervals (CI) and standard errors (SE) were calculated separately for each of the 5 multiply imputed datasets using the method by DeLong[5]. For pooling of AUC and confidence intervals, the function *pool_auc()* from package *psfmi* [7] was used, which applies Rubin’s rules after logarithmic transformation.

Sensitivity (Sens), specificity (Spec), positive predictive value (PPV), negative predictive value (NPV) were calculated based on the Youden point using the function *coords()* in the package *pROC* [5]. For estimation of confidence intervals, 2000-fold ordinary nonparametric bootstrapping was performed using the function *ci.coords()* from the package *pROC* [4]. The standard error was calculated from the 95% CI by the following formula SE = (empirical 97.5% quantile - empirical 2.5% quantile)/3.92. As for AUC, the function *pool_auc()* was used to calculate pooled estimates of the threshold-dependent metrics (Sens, Spec, PPV, NPV) as well as 95%CI.


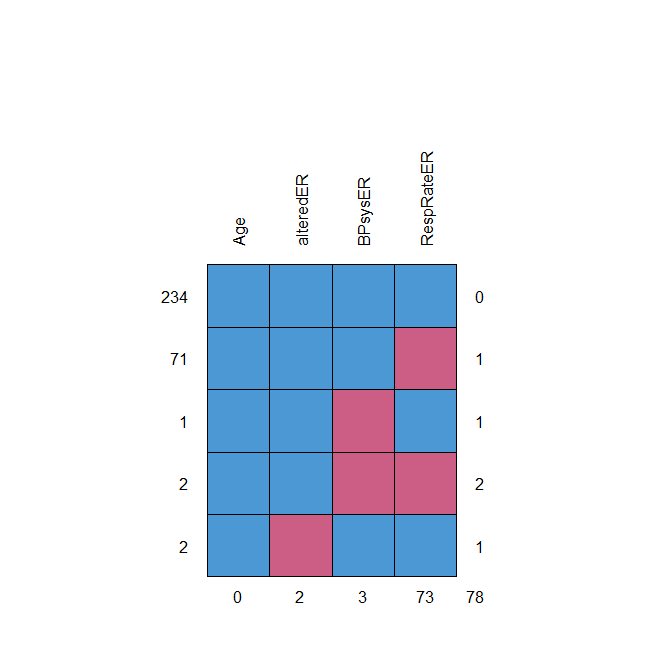


**Fig. S1 Missing data pattern for CRB-65**

Blue fields indicating complete data and red fields indicating missing data for each variable. Abbreviations: alteredER - confusion, BPsysER - systolic blood pressure, RespRateER - respiratory rate.


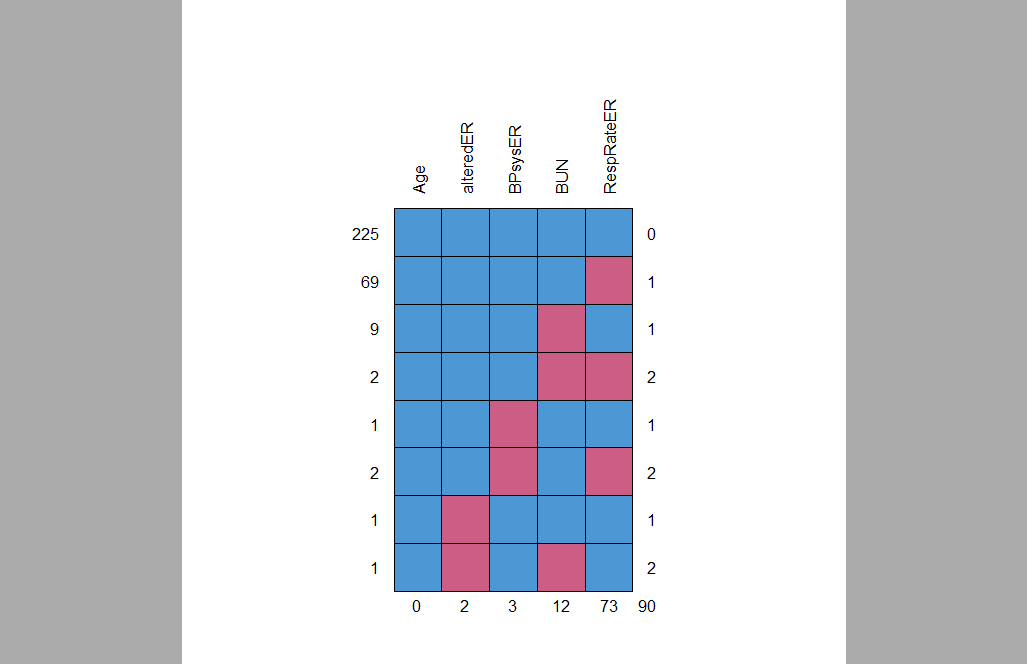


Fig. 2 Missing data pattern for CURB-65

Blue fields indicating complete data and red fields indicating missing data for each variable. Abbreviations: alteredER - confusion, BPsysER - systolic blood pressure, BUN – blood urea nitrogen, RespRateER - respiratory rate.


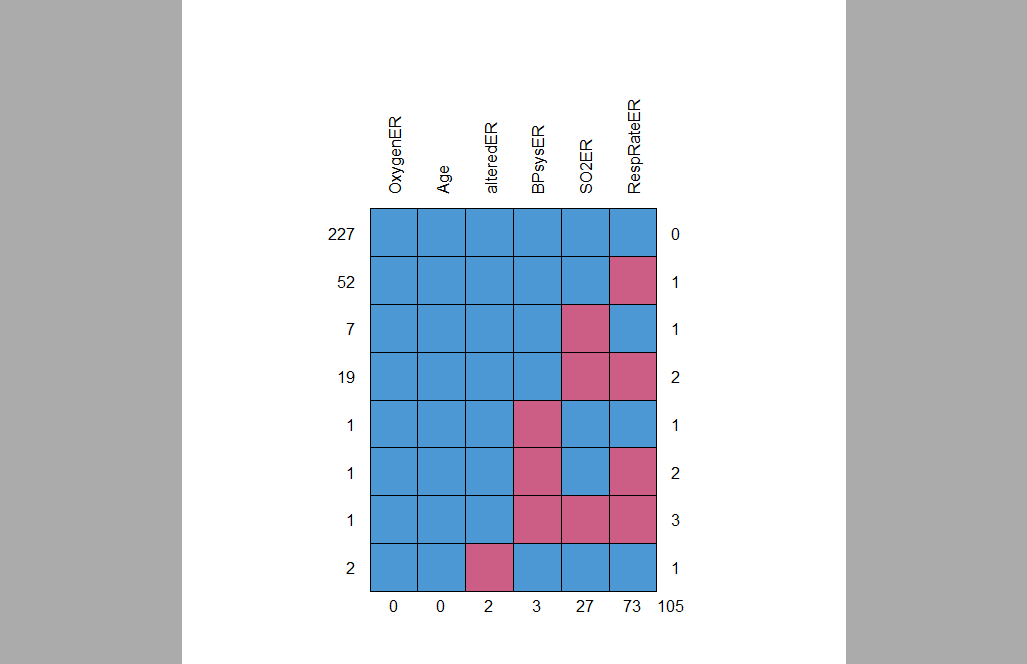


**Fig. S3 Missing data pattern for DS-CRB-65**

Blue fields indicating complete data and red fields indicating missing data for each variable. Abbreviations: OxygenER: oxygen flow, alteredER - confusion, BPsysER - systolic blood pressure, SO2ER – oxygen saturation, RespRateER - respiratory rate.


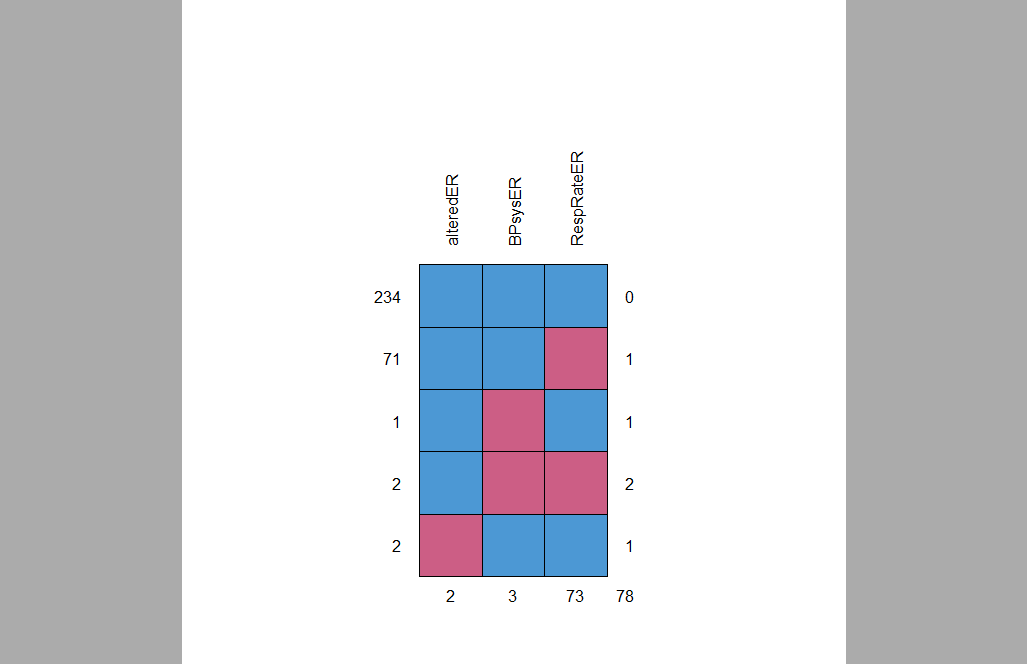


**Fig. S4 Missing data pattern for qSOFA**

Blue fields indicating complete data and red fields indicating missing data for each variable. Abbreviations: alteredER - confusion, BPsysER - systolic blood pressure, RespRateER - respiratory rate.


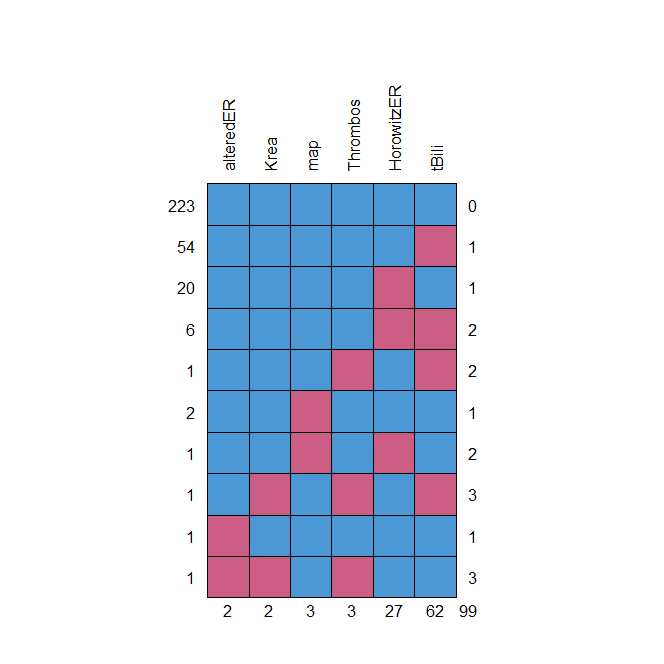


**Fig. S5 Missing data pattern for SOFA**

Blue fields indicating complete data and red fields indicating missing data for each variable. Abbreviations: alteredER - confusion, Krea – creatinine, map - mean arterial pressure, Thrombos – platelet count, HorowitzER – Horovitz index, tBili – total bilirubin.


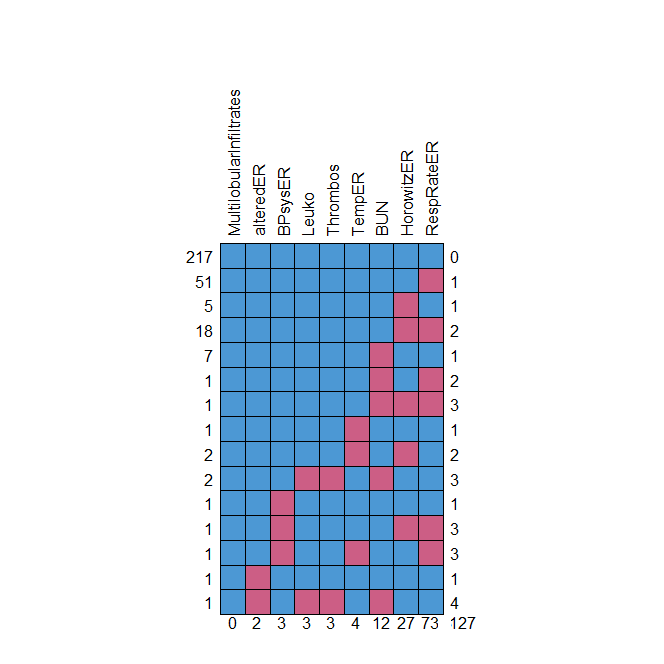


**Fig. S6 Missing data pattern for IDSA/ATS minor criteria**

Blue fields indicating complete data and red fields indicating missing data for each variable. Abbreviations: alteredER - confusion, BPsysER – systolic blood pressure, Leuko – white blood cell count, Thrombos – platelet count, TempER – temperature, BUN – blood urea nitrogen, HorowitzER – Horovitz index, RespRateER – respiratory rate.


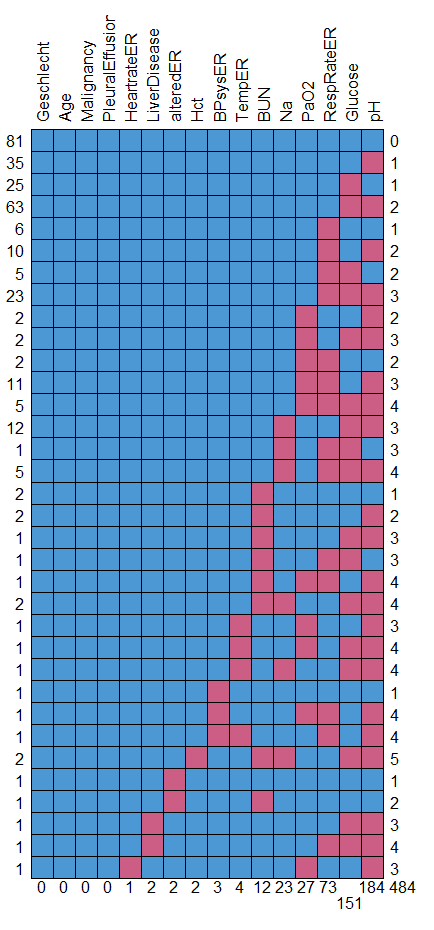


**Fig. S7 Missing data pattern for PSI**

Blue fields indicating complete data and red fields indicating missing data for each variable. Abbreviations: Geschlecht – sex, alteredER - confusion, Hct – hematocrit, BPsysER - systolic blood pressure, TempER – temperature, BUN – blood urea nitrogen, Na – sodium, RespRateER - respiratory rate.


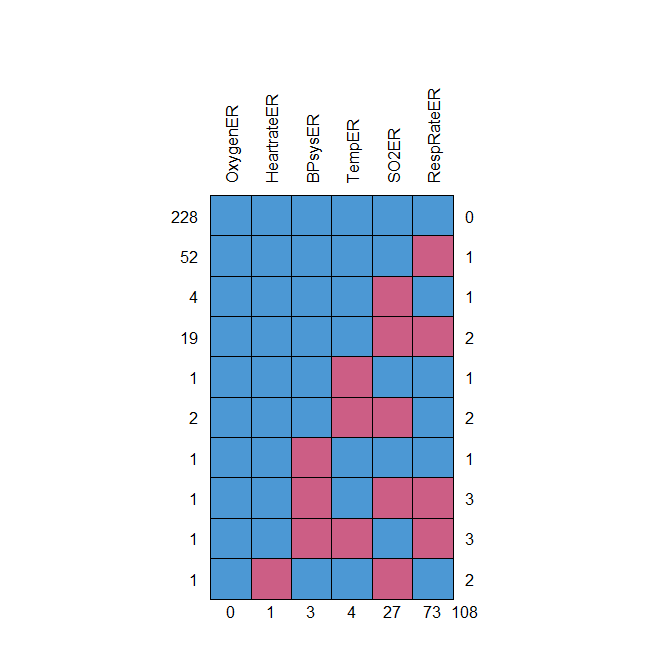


**Fig. S8 Missing data pattern for NEWS-2**

Blue fields indicating complete data and red fields indicating missing data for each variable. Abbreviations: OxygenER – oxygen flow, BPsysER - systolic blood pressure, TempER – temperature, SO2ER – oxygen saturation, RespRateER - respiratory rate.


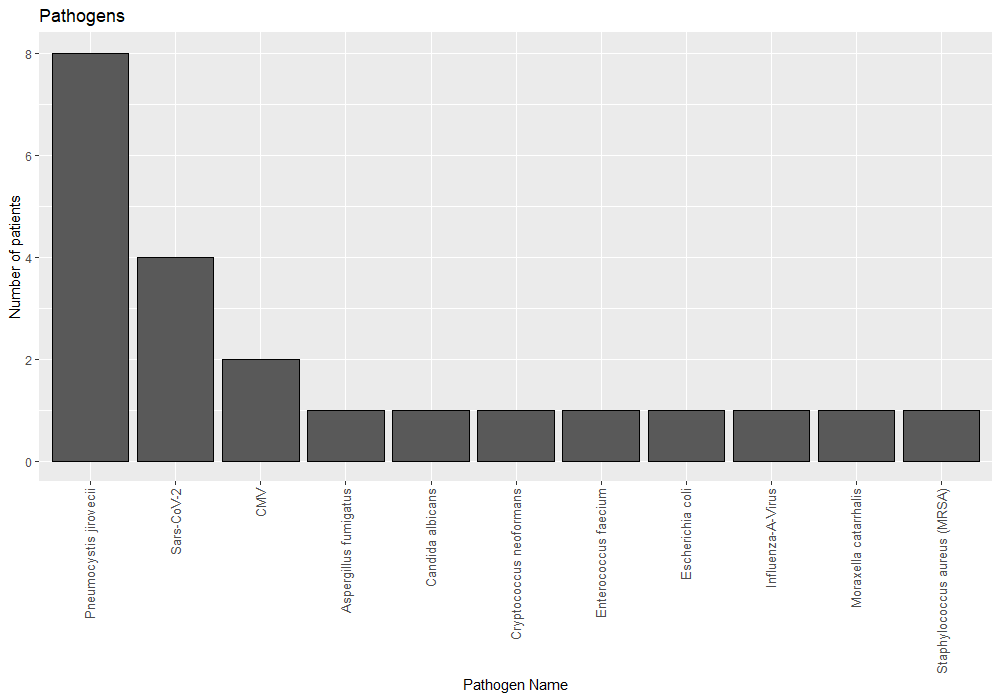


**Fig. S9 Causative pathogens isolated in the first year after transplantation in kidney transplant recipients presenting with the first episode of community acquired pneumonia**

SARS-CoV-2 - severe acute respiratory syndrome coronavirus 2, CMV - cytomegalovirus, MRSA - methicillin resistant staphylococcus aureus, MSSA - methicillin susceptible staphylococcus aureus, HSV - herpes simplex virus, RSV - respiratory syncytial virus.


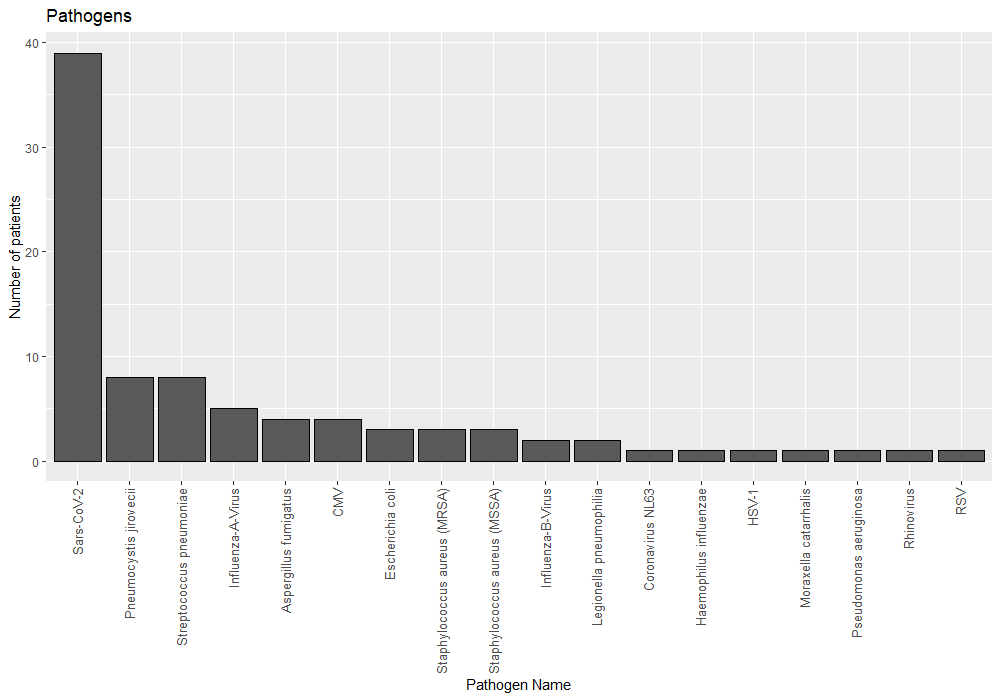


**Fig. S10 Causative pathogens isolated in the second and following years after transplantation in kidney transplant recipients presenting with the first episode of community acquired pneumonia**

SARS-CoV-2 - severe acute respiratory syndrome coronavirus 2, CMV - cytomegalovirus, MRSA - methicillin resistant staphylococcus aureus, MSSA - methicillin susceptible staphylococcus aureus, HSV - herpes simplex virus, RSV - respiratory syncytial virus.


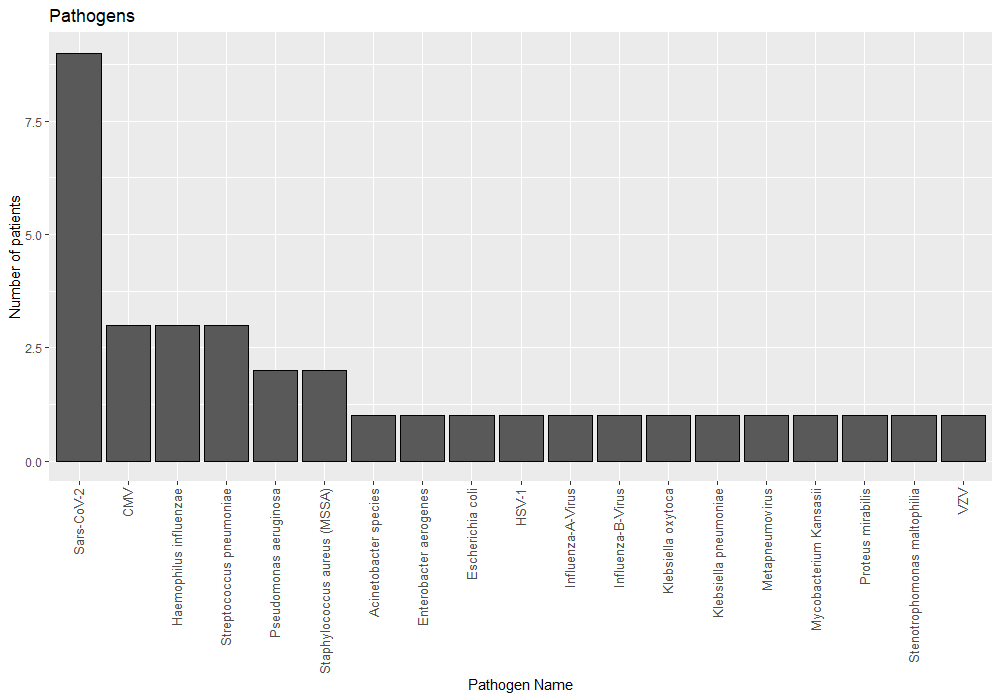


**Fig. S11 Causative pathogens isolated in kidney transplant recipients presenting with a recurrent episode of community acquired pneumonia**

SARS-CoV-2 - severe acute respiratory syndrome coronavirus 2, CMV - cytomegalovirus, MRSA - methicillin resistant staphylococcus aureus, MSSA - methicillin susceptible staphylococcus aureus, HSV - herpes simplex virus, VZV - varizella zoster virus.

**Table S1 Detailed analysis of 6 patients presenting with cytomegalovirus (CMV) pneumonia as the first community acquired pneumonia^1)^**

| **Patient No.** | **Time since transplantation** | **CMV risk status (donor CMV IgG -> recipient CMV IgG)** | **Other risk factors** | **Unfavorable outcome** | **Extrapulmonary CMV**  **manifestation** |
| --- | --- | --- | --- | --- | --- |
| 1 | 9 months | pos -> neg | valganciclovir prophylaxis discontinued 2 months before | - | colitis, hepatitis. viremia |
| 2 | 2.5 years | pos -> pos | low-replicative CMV viremia 2 years ago | - | - |
| 3 | 4.8 years | neg -> neg | possible primary infection due to work with children | - | gastritis, viremia |
| 4 | 15.7 years | pos -> neg | - | - | colitis, viremia |
| 5 | 4.2 years | neg -> neg | - | severe pneumonia | viremia |
| 6 | 8 months | neg -> neg | valganciclovir prophylaxis discontinued 1 month before | - | - |

^1)^Time since transplantation, CMV risk status, other risk factors for CMV infection as well outcome and extrapulmonary CMV manifestations are summarized.

**Item S7 Outcomes for pneumocystis jirovecii pneumonia and negative predictive value of lactate dehydrogenase (LDH) for ruling out PjP in kidney transplant recipients**

For patients with pneumocystis jirovecii (PjP), 2 out of 16 patients (12.5%) had severe pneumonia, whereas 15.5 % had severe pneumonia in the non-PjP group (no significant difference). PjP pneumonia occurred earlier than in patients with non PjP pneumonia (median time since transplantation 1.1 vs. 4.1 years, p=0.013). LDH was increased in all patients with PjP pneumonia above the cutoff of 250 U/mL, but it was not significantly higher than in the non-PjP group. Consequently, LDH had a sensitivity and negative predictive value of 1.0 in this small cohort.

**Table S2 Frequency of in-hospital mortality and severe pneumonia depending on score value for 8 different risk prognostication scores in kidney transplant recipients hospitalized for community acquired pneumonia^2)^**

| **Score** | **Value** | **Total patients** | **Patients died (%)** | **Patients with severe pneumonia (%)** |
| --- | --- | --- | --- | --- |
| **CRB-65** | 0 | 147 | 4 (3%) | 11 (7%) |
|  | 1 | 116 | 7 (6%) | 27 (23%) |
|  | 2 | 39 | 3 (8%) | 6 (15%) |
|  | 3 | 8 | 2 (25%) | 4 (50%) |
| **CURB-65** | 0 | 35 | 0 (0%) | 0 (0%) |
|  | 1 | 127 | 4 (3%) | 11 (9%) |
|  | 2 | 106 | 7 (7%) | 27 (25%) |
|  | 3 | 34 | 3 (9%) | 6 (18%) |
|  | 4 | 8 | 2 (25%) | 4 (50%) |
| **DS-CRB-65** | ≤ 1 | 118 | 1 (1%) | 3 (3%) |
|  | 2 | 101 | 7 (7%) | 20 (20%) |
|  | 3 | 71 | 6 (8%) | 18 (25%) |
|  | 4 | 15 | 0 (0%) | 3 (20%) |
|  | 5 | 5 | 2 (40%) | 4 (80%) |
| **qSOFA** | 0 | 197 | 4 (2%) | 15 (8%) |
|  | 1 | 102 | 8 (8%) | 24 (24%) |
|  | 2 | 10 | 3 (30%) | 8 (80%) |
|  | 3 | 1 | 1 (100%) | 1 (100%) |
| **SOFA** | ≤1 | 95 | 0 (0%) | 2 (2%) |
|  | 2 | 87 | 3 (3%) | 7 (8%) |
|  | 3 | 47 | 4 (9%) | 11 (23%) |
|  | 4 | 41 | 3 (7%) | 9 (22%) |
|  | 5 | 25 | 1 (4%) | 7 (28%) |
|  | 6 | 5 | 1 (20%) | 3 (60%) |
|  | 7 | 3 | 0 (0%) | 2 (67%) |
|  | 8 | 2 | 1 (50%) | 2 (100%) |
|  | 9 | 1 | 1 (100%) | 1 (100%) |
|  | ≥10 | 4 | 2 (50%) | 4 (100%) |
| **IDSA/ATS**  **minor criteria** | 0 | 22 | 0 (0%) | 0 (0%) |
|  | 1 | 99 | 1 (1%) | 6 (6%) |
|  | 2 | 122 | 7 (6%) | 18 (15%) |
|  | 3 | 48 | 4 (8%) | 16 (33%) |
|  | 4 | 15 | 4 (27%) | 7 (47%) |
|  | ≥5 | 4 | 0 (0%) | 1 (25%) |
| **PSI** | <60 | 15 | 0 (0%) | 1 (7%) |
|  | 60-89 | 85 | 2 (2%) | 7 (8%) |
|  | 90-119 | 83 | 4 (5%) | 10 (12%) |
|  | 120-149 | 82 | 3 (4%) | 17 (21%) |
|  | 150-179 | 34 | 4 (12%) | 8 (24%) |
|  | ≥180 | 11 | 3 (27%) | 5 (45%) |
| **NEWS-2** | 0 | 46 | 1 (2%) | 1 (2%) |
|  | 1 | 46 | 1 (2%) | 1 (2%) |
|  | 2 | 46 | 0 (0%) | 2 (4%) |
|  | 3 | 42 | 1 (2%) | 5 (12%) |
|  | 4 | 31 | 2 (6%) | 6 (19%) |
|  | 5 | 25 | 1 (4%) | 5 (20%) |
|  | 6 | 30 | 3 (10%) | 9 (30%) |
|  | 7 | 14 | 0 (0%) | 4 (29%) |
|  | 8 | 16 | 5 (31%) | 7 (44%) |
|  | 9 | 5 | 0 (0%) | 3 (60%) |
|  | 10-13 | 7 | 1 (14%) | 3 (43%) |
|  | 14-15 | 2 | 1 (50%) | 2 (100%) |

^2)^ Results are shown for one out of five multiply imputed datasets.

**Table S3 Univariable and multivariable logistic regression for variables included within eight risk scores with the primary endpoint - severe pneumonia**

| **Variable** | **Univariable analysis**    **median p-value** | **Multivariable analysis**    **median p-value** |
| --- | --- | --- |
| Age | 0.110 | 0.692 |
| Female sex | 0.79 | 0.334 |
| Altered mental status | **<0.001** | 0.146 |
| Temperature | 0.740 | 0.485 |
| Heartrate | **0.017** | 0.187 |
| Systolic BP | 0.558 | 0.667 |
| Diastolic BP | 0.058 | 0.113 |
| Respiratory rate | **0.015** | 0.310 |
| SpO_2_ | **<0.001** | 0.969 |
| Oxygen | **<0.001** | 0.964 |
| PaO_2_ | **<0.001** | 0.955 |
| Horovitz index | **<0.001** | 0.568 |
| Na+ | **0.014** | 0.270 |
| pH | 0.199 | 0.734 |
| Glucose | **0.020** | 0.292 |
| BUN | **<0.001** | **<0.001** |
| Creatinine | **<0.001** | 0.861 |
| Bilirubin | **0.012** | 0.018 |
| Hematocrit | 0.493 | 0.702 |
| Platelet count | 0.309 | 0.499 |
| WBC | 0.586 | 0.749 |
| Malignancy | 0.980 | 0.497 |
| Liver disease | 0.738 | 0.809 |
| Congestive heart failure | **<0.001** | 0.231 |
| Cerebrovascular disease | 0.712 | 0.768 |
| Pleural effusion | **0.008** | 0.659 |
| Multilobular infiltrates | **0.009** | **0.073** |

**Table S4 Complete case analysis of ROC analysis for eight different risk prognostication scores predicting the occurrence of in-hospital mortality in 310 kidney transplant recipients with community acquired pneumonia^3)^**

| **Score** | **AUC** | **Sens** | **Spec** | **PPV** | **NPV** | **Youden** | **Cutoff** |
| --- | --- | --- | --- | --- | --- | --- | --- |
| **CRB-65** | 0.665 (0.521 - 0.809) | 0.667 (0.133 - 0.933) | 0.557 (0.466 - 0.991) | 0.111 (0.078 - 0.600) | 0.964 (0.943 - 0.992) | 1.286 | 0.5 |
| **CURB-65** | 0.698 (0.560 - 0.836) | 0.643 (0.214 - 1.000) | 0.597 (0.133 - 0.986) | 0.118 (0.071 - 0.500) | 0.967 (0.949 - 1.000) | 1.315 | 1.5 |
| **DS-**  **CRB-65** | 0.712 (0.601 - 0.823) | 0.933 (0.533 - 1.000) | 0.443 (0.373 - 0.778) | 0.108 (0.090 - 0.187) | 0.989 (0.958 - 1.000) | 1.383 | 1.5 |
| **qSOFA** | 0.742 (0.609 - 0.875) | 0.733 (0.267 - 0.933) | 0.690 (0.626 - 0.977) | 0.143 (0.100 - 0.556) | 0.974 (0.950 - 0.994) | 1.427 | 0.5 |
| **SOFA** | 0.813 (0.721 - 0.905) | 0.867 (0.533 - 1.000) | 0.654 (0.365 - 0.947) | 0.157 (0.102 - 0.421) | 0.986 (0.966 - 1.000) | 1.525 | 2.5 |
| **PSI** | 0.730 (0.548 - 0.913) | 0.700 (0.400 - 1.000) | 0.845 (0.394 - 0.972) | 0.381 (0.174 - 0.778) | 0.952  (0.914 - 1.000) | 1.487 | 134.5 |
| **IDSA/ATS Minor** | 0.736 (0.620 - 0.853) | 0.929 (0.357 - 1.000) | 0.458 (0.379 - 0.970) | 0.108 (0.089 - 0.500) | 0.988 (0.955 - 1.000) | 1.389 | 1.5 |
| **NEWS-2** | 0.748 (0.608 - 0.887) | 0.800 (0.400 - 1.000) | 0.666 (0.478 - 0.938) | 0.151 (0.103 - 0.400) | 0.977 (0.955 - 1.000) | 1.464 | 3.5 |

^3)^ Variables were assessed up to 48 hours after hospital admission, but always before an endpoint was reached.

**Table S5 Complete case analysis of ROC analysis for eight different risk prognostication scores predicting the occurrence of primary endpoint (composite endpoint of invasive mechanical ventilation, vasopressor treatment, dialysis, or in-hospital mortality) in 310 kidney transplant recipients with community acquired pneumonia^4)^**

| **Score** | **AUC** | **Sens** | **Spec** | **PPV** | **NPV** | **Youden** | **Cutoff** |
| --- | --- | --- | --- | --- | --- | --- | --- |
| **CRB-65** | 0.674 (0.593 - 0.755) | 0.767 (0.628 - 0.884) | 0.565 (0.497 - 0.639) | 0.286 (0.238 - 0.333) | 0.916 (0.869 - 0.957) | 1.336 | 0.5 |
| **CURB-65** | 0.724 (0.650 - 0.798) | 0.781 (0.659 - 0.902) | 0.614 (0.544 - 0.685) | 0.311 (0.260 - 0.886) | 0.927 (0.886 - 0.966) | 1.397 | 1.5 |
| **DS-**  **CRB-65** | 0.771  (0.706 - 0.837) | 0.954 (0.628 - 1.000) | 0.500 (0.424 - 0.821) | 0.311 (0.277 - 0.469) | 0.978 (0.899 - 1.000) | 1.453 | 1.5 |
| **qSOFA** | 0.744 (0.663 - 0.824) | 0.698 (0.558 - 0.837) | 0.738 (0.675 - 0.801) | 0.377 (0.304 - 0.453) | 0.915 (0.879 - 0.950) | 1.436 | 0.5 |
| **SOFA** | 0.826 (0.762 - 0.890) | 0.837 (0.698 - 0.930) | 0.722 (0.656 - 0.794) | 0.419 (0.357 - 0.500) | 0.949 (0.913 - 0.979) | 1.559 | 2.5 |
| **PSI** | 0.744 (0.631 - 0.857) | 0.724 (0.448 - 0.931) | 0.750 (0.519 - 0.942) | 0.618 (0.488 - 0.826) | 0.828 (0.742 - 0.930) | 1.462 | 107 |
| **IDSA/ATS Minor** | 0.778 (0.706 - 0.849) | 0.829 (0.463 - 0.976) | 0.551 (0.438 - 0.909) | 0.325 (0.268 - 0.595) | 0.931 (0.874 - 0.989) | 1.417 | 1.5 |
| **NEWS-2** | 0.824 (0.759 - 0.890) | 0.861 (0.674 - 0.977) | 0.694 (0.530 - 0.842) | 0.394 (0.319 - 0.528) | 0.955 (0.913 - 0.991) | 1.542 | 3.5 |

^4)^ Variables were assessed up to 48 hours after hospital admission, but always before an endpoint was reached.

**Table S6 Comparison of in-hospital mortality or severe pneumonia in patients with COVID-19 vs. Non-COVID-19 pneumonia by ROC analysis for eight risk prognostication scores in 310 kidney transplant recipients with community acquired pneumonia**

| Score | COVID-19  In-hospital Death  AUC | Non-COVID-19  In-hospital Death  AUC | COVID-19  Severe  pneumonia  AUC | Non-COVID-19  Severe pneumonia  AUC |
| --- | --- | --- | --- | --- |
| CRB-65 | 0.651 (0.446 - 0.812) | 0.689 (0.426 - 0.869) | 0.673 (0.504 - 0.807) | 0.640 (0.523 - 0.742) |
| CURB-65 | 0.693 (0.490 - 0.841) | 0.625 (0.241 - 0.897) | 0.766 (0.400 - 0.941) | 0.679 (0.569 - 0.773) |
| DS-  CRB-65 | 0.604 (0.417 - 0.765) | 0.758 (0.563 - 0.884) | 0.669 (0.500 - 0.803) | 0.734 (0.631 - 0.817) |
| qSOFA | 0.692 (0.480 -   0.845) | 0.742 (0.487 -  0.897) | 0.733 (0.554 - 0.858) | 0.695 (0.571 - 0.796) |
| SOFA | 0.680 (0.497 -  0.820) | 0.914 (0.798 - 0.966) | 0.804 (0.648 - 0.901) | 0.843 (0.749 - 0.906) |
| PSI | 0.663 (0.431 -  0.837) | 0.825 (0.553 - 0.947) | 0.744 (0.549 - 0.875) | 0.676 (0.538 - 0.788) |
| ATS Minor | 0.520 (0.361 - 0.676) | 0.815 (0.557 -  0.939) | 0.655 (0.493 -  0.788) | 0.732 (0.627 - 0.816) |
| NEWS-2 | 0.685 (0.472 - 0.841) | 0.787 (0.494 - 0.933) | 0.787 (0.614 -  0.895) | 0.790 (0.684 - 0.867) |

**Supplementary References**

1. Krüger S, Ewig S, Papassotiriou J, Kunde J, Marre R, von Baum H, et al. Inflammatory parameters predict etiologic patterns but do not allow for individual prediction of etiology in patients with CAP-Results from the German competence network CAPNETZ. 2009.
2. Rice TW, Wheeler AP, Bernard GR, Hayden DL, Schoenfeld DA, Ware LB et al. National Institutes of Health, National Heart, Lung, and Blood Institute ARDS Network. Comparison of the SpO2/FIO2 ratio and the PaO2/FIO2 ratio in patients with acute lung injury or ARDS. Chest. 2007 Aug;132(2):410-7. doi: 10.1378/chest.07-0617. Epub 2007 Jun 15. PMID: 17573487.
3. Inker LA, Eneanya ND, Coresh J, Tighiouart H, Wang D, Sang Y, et al. New Creatinine- and Cystatin C–Based Equations to Estimate GFR without Race. New England Journal of Medicine [Internet]. Massachusetts Medical Society; 2021 ;385:1737–49. <https://www.nejm.org/doi/10.1056/NEJMoa2102953>
4. van Buuren S, Groothuis-Oudshoorn K. mice: Multivariate Imputation by Chained Equations in R. J Stat Softw [Internet]. 2011 ;45:1–67. https://www.jstatsoft.org/index.php/jss/article/view/v045i03
5. Robin X, Turck N, Hainard A, Tiberti N, Lisacek F, Sanchez JC, et al. pROC: An open-source package for R and S+ to analyze and compare ROC curves. BMC Bioinformatics [Internet]. BioMed Central; 2011 ;12:1–8. <https://bmcbioinformatics.biomedcentral.com/articles/10.1186/1471-2105-12-77>
6. DeLong ER, DeLong DM, Clarke-Pearson DL. Comparing the Areas under Two or More Correlated Receiver Operating Characteristic Curves: A Nonparametric Approach. Biometrics. JSTOR; 1988;44:837.
7. Heymans M. psfmi: Prediction Model Pooling, Selection and Performance Evaluation Across Multiply Imputed Datasets. R package version 1.1.0, https://mwheymans.github.io/psfmi/. 2022.
